# Supplementary material for: Characterization of Cronobacter sakazakii and Cronobacter malonaticus Strains Isolated from Powdered Dairy Products Intended for Consumption by Adults and Older Adults
Source: Microorganisms. 2023 Nov 23;11(12):2841. doi: 10.3390/microorganisms11122841 (PMC10745994; doi:10.3390/microorganisms11122841)
Supplement: Supplementary file 1 [file microorganisms-11-02841-s001.zip › microorganisms-2665715-supplementary.pdf]

Supplementary Table S1: Process data of genomic sequencing of the strains studied.

ID	Avg. Coverage	AVg. Read Length	No. of Contigs	N50	Genome Size /Mb)	No. of Reads
510299	77	248	464	232,579	5.03	1,345,648
510300	63	263	127	167,359	4.75	1,050,716
510178-22	46	155	203	69,168	4.5	1,152,347
510177-22	68	153	1,822	78,588	5.4	1,470,178
510441	56	229	1,983	246,448	5.6	1,065,592
